# *Drosophila* Arc1 is not required for male fertility or sperm competition success

**DOI:** 10.17912/micropub.biology.001053

**Published:** 2023-11-27

**Authors:** Kathleen Gordon, Patrick Gonzales, Caroline Lee, Jeremy Marcin, Yoko Takashima, Brian Lazzaro, Mariana Wolfner

**Affiliations:** 1 Department of Entomology, Cornell University, Ithaca, New York, United States; 2 Department of Molecular Biology and Genetics, Cornell University, Ithaca, New York, United States

## Abstract

Activity-regulated cytoskeleton associated protein (Arc1), which is required for synaptic plasticity and metabolism in *Drosophila* , self-assembles into capsid-like structures that transport mRNAs in extracellular vesicles. In addition to expression in the brain and nervous system, *Arc1* is expressed in the male accessory glands, an endothelial tissue that produces male seminal proteins and exosomes that impact male fertility. We thus hypothesized that *Arc1* might impact male fertility. We measured the fertility, mating latency, mating duration, and sperm competition performance of *Arc1* males relative to controls and found no evidence that *Arc1* is required for any of these measures of male fertility.

**Figure 1. Arc1 is not required for male fertility, copulation, or sperm competition success:**
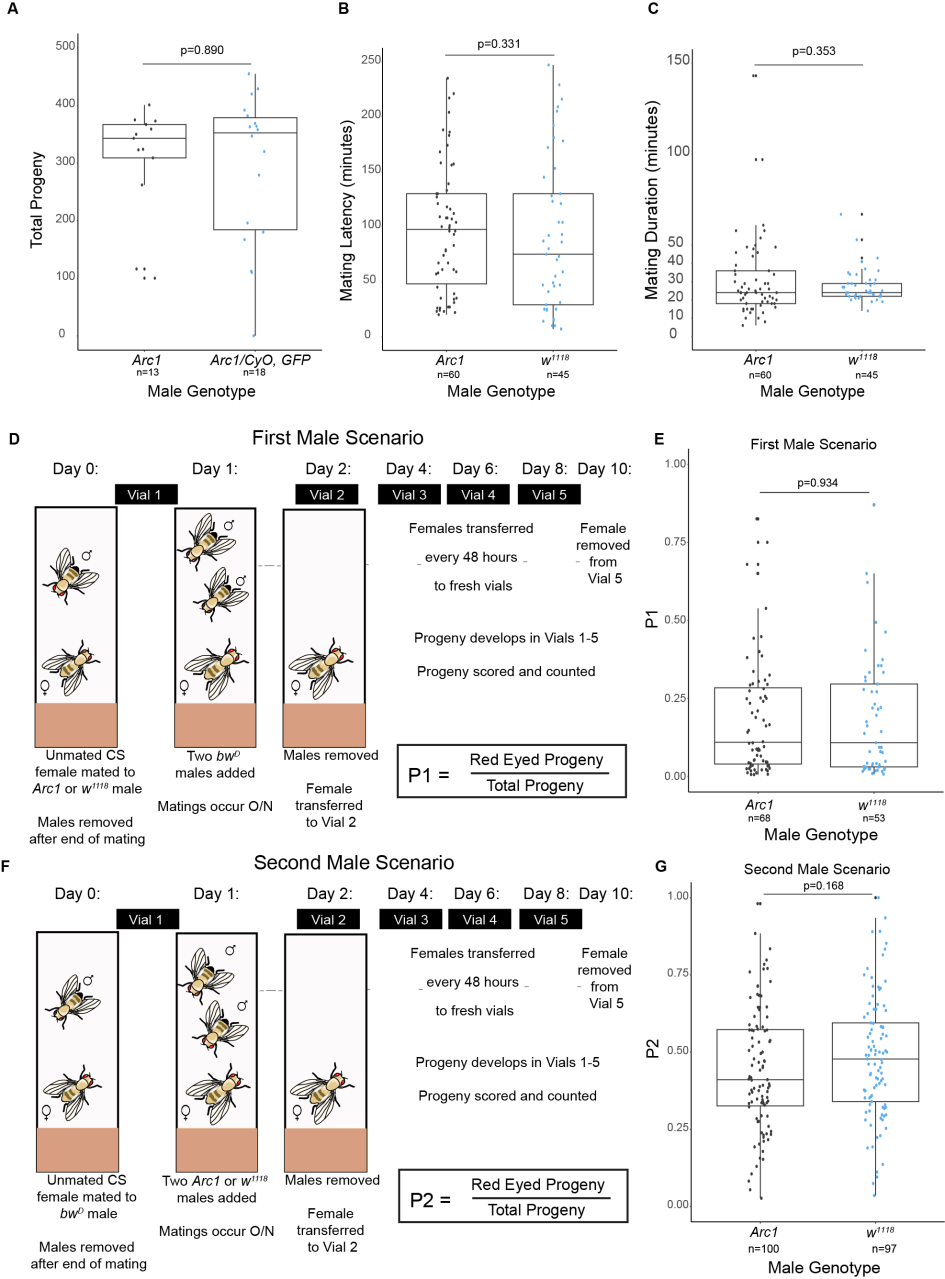
**A)** Total progeny produced by homozygous *Arc1* mutants (n=13) and heterozygous *Arc1/CyO, GFP* control males (n=18). Wilcoxon rank sum test: *p* =0.890). **B)** Mating latency of *Arc1* mutants (n=60) and *w ^1118^* controls (n=45). Welch’s t-test: *p* =0.311 **C)** Mating duration of *Arc1* mutants (n=60) and *w ^1118^* controls (n=45). Welch’s t-test: *p* =0.353. **D)** First male experimental set up, where *Arc1* mutants or *w ^1118^* control males were first to mate with wild-type females, followed by *bw ^D^* males. **E)** Paternal share (P1) of *Arc1* mutants (n=68) and *w ^1118^* controls (n=53) in first male scenario. Two way t-test: *p* =0.934 **F)** Second male experimental set up, where *bw ^D^* males were first to mate with wild-type females, followed by *Arc1* mutants or *w ^1118^* control males. **G)** Paternal share (P2) of *Arc1* mutants (n=100) and *w ^1118^* controls (n=97) in second male scenario. Two way t-test: *p* =0.168.

## Description

Activity-regulated cytoskeleton associated protein (ARC) is an immediate-early gene that responds to neuronal activity (reviewed in Shepherd & Bear, 2011) . Mammalian ARC is required for synaptic plasticity and long term memory, and mutations in human Arc are associated with neuronal disorders like schizophrenia, Alzheimer’s, and autism spectrum disorders (reviewed in Shepherd & Bear, 2011) . Both mammalian ARC and *Drosophila* Arc1 contain retroviral group-specific, antigen-like amino acid sequences that are thought to derive from Ty3 retrotransposons (Cottee et al., 2020) . Using a Gag-like protein, both mammalian ARC and *Drosophila* Arc1 form capsids containing their mRNA that are packaged inside extracellular vesicles which can be secreted from neurons (Ashley et al., 2018; Pastuzyn et al., 2018) . In *Drosophila melanogaster* , Arc1 is associated with synapse maturation and plasticity (Ashley et al., 2018) . Additionally, *Arc1* mutants have altered metabolism, high levels of fat storage, and are resistant to starvation (Keith et al., 2021; Mattaliano et al., 2007; Mosher et al., 2015) . Furthermore, Keith et al. (2021) showed that *Arc1* interacts with the microbiome to regulate growth and metabolic homeostasis.

Although mammalian Arc and *Drosophila* Arc1 are primarily expressed in the brain and nervous system, both also show reproductive tract expression. Mammalian Arc is expressed in testes (Shepherd & Bear, 2011) and Arc1 is expressed in the *Drosophila* male accessory gland (Leader et al., 2018) . The male accessory gland is an endothelial secretory tissue that produces seminal proteins and exosomes that are transferred to females during mating (reviewed in Avila et al., 2011; Wilson et al., 2017). These male accessory gland molecules then regulate female post-mating behaviors such as refractoriness to remating and sustained egg production (reviewed in Avila et al., 2011; Wilson et al., 2017). However, it is not known whether Arc plays a role in male reproductive success in either mammals or insects.

We found that homozygous mutant *w ^1118^ ; Arc1 ^E8^* ( *Arc1* ) males sire a normal number of progeny, indicating that *Arc1* is not required for male fertility ( Figure 1A : Wilcoxon Rank Sum Test, *p* =0.890). However, multiple other traits impact male reproductive success, including the relative success of a male in competition with other males. Because female *Drosophila melanogaster* can mate with multiple mates and store sperm for days to weeks, male ejaculates can compete for reproductive success within the female, as reflected in paternal progeny proportion (Bloch Qazi et al., 2003; Giardina et al., 2017; Neubaum & Wolfner, 1999) . Seminal fluid components derived from the male accessory gland, as well as sperm length, number, and speed, are known to influence paternity proportion (Castillo & Moyle, 2014; Chow et al., 2013; Clark et al., 1995; Clark & Begun, 1998; Fiumera et al., 2005, 2007; Fricke et al., 2009; Zhang et al., 2013) . Furthermore, in response to competition with a rival, male *D. melanogaster* can decrease mating latency and increase mating duration, which can ultimately lead to an increase in paternal progeny proportion (Bretman et al., 2009) . Changes in mating duration in competitive environments are linked to memory processing through activity in the mushroom body (Rouse et al., 2018) . Given the role of Arc1 in synapse plasticity (Ashley et al., 2018) and its expression in male accessory gland (Leader et al., 2018) , we hypothesized that dArc1 could affect mating duration and ultimately sperm competition success.

To test our hypothesis, we observed matings between wild-type Canton S (CS) females and *Arc1* mutant or *w ^1118^* control males, and measured both mating latency and mating duration. We found no difference in mating latency ( Figure 1B : Welch’s t-test, *p=* 0.331) or mating duration ( Figure 1C : Welch’s t-test, *p=* 0.353) between *Arc1* mutants and *w ^1118^* controls. We next assayed the sperm competition success of *Arc1* mutants in two different experimental set-ups, competing *Arc1* or *w ^1118^* control males against males with a dominant allele of the eye color gene, *brown* ( *bw ^D^* ). In the first male scenario, *Arc1* or *w ^1118^* control males were mated to a female first, followed by the second mate: *bw ^D^* males ( Figure 1D ). In the second male scenario, females were first mated to a *bw ^D^* male, followed by either an *Arc1* or a *w ^1118^* control male ( Figure 1F ). In both competitive experimental set-ups, the paternity of each progeny could be determined by eye color: all progeny of *bw ^D^* males and the CS females have brown eyes while all progeny of *Arc1* or *w ^1118^* males and the CS female have wildtype eyes. For the first male scenario, we calculated the paternity proportion (P1) of the first male ( *Arc1* or *w ^1118^* ) as the total number of first male progeny divided by the total number of progeny. For the second male scenario, we calculated the paternity proportion (P2) of the second male ( *Arc1* or *w ^1118^* ) as the total number of second male progeny divided by the total number of progeny. If Arc1 influences male sperm competition success, we would expect that *Arc1* mutant males will yield lower proportions of progeny relative to controls. Conversely, if Arc1 is not required for male sperm competitive success, we would expect *dArc1* mutant males to produce similar proportions of progeny as control males.

We found no difference in P1 between *Arc1* mutants and *w ^1118^* controls ( Figure 1E, two sample t -test, *p* =0.934). Similarly, we found no difference in P2 between *Arc1* mutants and *w ^1118^* controls ( Figure 1G, two sample t -test, *p* =0.168). Thus, despite the expression of *Arc1* in both the nervous system and the male reproductive tract, we found no evidence of fertility defects, or impaired mating latency, duration, or sperm competition success in *Arc1* mutant males.

These negative results indicate that Arc1 is not required for *Drosophila* male reproductive success. However, the role of *Drosophila* Arc1 and mammalian ARC in male reproductive tissues remains to be elucidated. Future studies to define the localization of Arc1 in the male accessory gland or comparative transcriptomics and proteomics on *Arc1* mutant versus control accessory glands could help identify a functional role for Arc1 in male reproductive health.

## Methods

***Fly Husbandry and Genotypes***

All flies were raised on cornmeal-sucrose media (weight by volume in 1 L of H _2_ O: 0.7% agar, 6% Brewer’s yeast, 6% cornmeal, and 4% sucrose with 26.5 mL of 100g Tegosept in 95% ethanol and 12 mL mixture of 0.04% phosphoric acid and 0.4% propionic acid to inhibit microbial growth). All flies were kept on a 12-hour light-dark cycle at room temperature (22-25 ^o^ C). *Arc1* mutants were a gift from Dr. Travis Thomson and are *w ^1118^ ; Arc1 ^E8^* or the balanced control, *w ^1118^ ; Arc1 ^E8^* /CyO, GFP (Ashley et al., 2018) . *w ^1118^* males were used as genetic background controls. The males with a dominant allele of *brown* (designated *bw ^D^* ) are *cn bw ^D^* . All females were wild-type Canton S.

***Male Fertility Test***

We tested the fertility of *w ^1118^ ; Arc1 ^E8^ (Arc1)* mutant males compared to heterozygous control males, *w ^1118^ ; Arc1 ^E8^* /CyO, GFP ( *Arc1/CyO, GFP)* . Single wild-type CS females (3-5 days old) were aspirated into a fresh food vial treated with dry yeast the night before the matings occurred. In the morning, single *Arc1* or *Arc1/CyO, GFP* males were aspirated into a vial containing a single female. Matings were observed and the start and end times of copulation were recorded. After mating, males were removed from the vial. Females were transferred to new food vials every 24 hours for 4 days, thereby creating four total vials per female. Progeny were allowed to develop to the adult stage, then counted. Total progeny is reported as the sum of progeny from all four vials per individual male. We compared the mean total progeny of *Arc1* and *Arc1/CyO, GFP* males by a Wilcoxon rank sum test.

***Mating Duration and Latency***

Mating latency is the length of time between introduction of the first male mate and initiation of copulation. Mating duration is the length of time from the initiation of copulation to the end of copulation. We used Welch’s t-test to compare both mean mating latency and mean mating duration between *Arc1* mutants and *w ^1118^* controls.

***Sperm Competition Experimental Design***

The sperm competition assay was based on (Chen et al., 2019) and (Chen et al., 2022) . The success of *Arc1* mutants was assayed in two different sperm competition experimental set-ups where *Arc1* males and *w ^1118^* controls were either the first or second male to mate with a single female. In the first male scenario, wild-type Canton S females were set up in single-pair matings with either *Arc1* or *w ^1118^* males on day 0 in vial 1. Copulations were observed and males were removed after mating ended. In the evening of day 1, two *bw ^D^* males were added to each vial and matings were allowed to occur overnight. In the morning of day 2, the *bw ^D^* males were removed and all females were transferred to vial 2. Each female was transferred every 48 hours for 8 days to a new vial for a total of 5 vials. Females were discarded on day 10 and all progeny were allowed to develop. Progeny were separated by eye color and counted. The paternity of the progeny was determined by eye color: progeny from *Arc1* or *w ^1118^* fathers had wild-type eyes, while progeny from *bw ^D^* fathers had brown eyes. In the second-male scenario, the same procedure was performed except *bw ^D^* males were first mated to Canton S females on day 0, while two *Arc1* or *w ^1118^* males were added in the evening of day 1.

For both sperm competition scenarios, the experiment was performed in two blocks. The experimenter counting progeny was blinded to the first or second male’s genotypic identity ( *Arc1* or *w ^1118^* ) by keeping the male identities in a key until progeny data were analyzed.

***Paternal Success of First Male (P1) and Second Male (P2)***

Females that did not survive the 10 days of the experiment or did not produce progeny from each of the two mates were discarded. In the first male scenario, paternal progeny proportion for the first male (P1) was calculated as the number of first male progeny divided by the total number of progeny. In the second male scenario, paternal progeny proportion for the second male (P2) was calculated as the number of second male progeny divided by the total number of progeny. Although both mating events took place in vial 1, there was more opportunity for fertilized eggs to be laid by the first male. Therefore, we discarded this vial for counts of P1 and P2. We compared mean P1 or P2 for *dArc1* mutants and *w ^1118^* controls using a two sample t-test.

***Data Analysis and Visualization***

All data was analyzed using R Studio Version 2023.06.0+421. All graphs were created using the package ggplot2. Experimental set-up figures were created and arranged with graphs using Adobe Illustrator. *Drosophila* cartoons are licensed under Creative Commons 3.0.
